# Association between the oxidative balance score and preserved ratio impaired spirometry in US adults: NHANES 2007–2012

**DOI:** 10.3389/fnut.2025.1551237

**Published:** 2025-08-06

**Authors:** Shengyuan Gu, Xinchao Du, Xiaoxia Gu

**Affiliations:** Department of Cardiology, Zhengzhou Central Hospital Affiliated to Zhengzhou University, Zhengzhou, Henan, China

**Keywords:** preserved ratio impaired spirometry, oxidative balance score, oxidative stress, dietary quality and lifestyle, antioxidant strategy

## Abstract

**Background and aims:**

The relationship between oxidative stress (OS) and preserved ratio impaired spirometry (PRISm) remains unclear. We aimed to utilize the oxidation balance score (OBS), a validated instrument for assessing the overall OS status, to investigate the association between OBS and PRISm.

**Methods:**

We included data from 7,180 participants in the National Health and Nutrition Examination Survey (NHANES). The OBS was calculated using 20 components of diet and lifestyle. Binary logistic regression analyses were conducted to investigate the association between OBS and PRISm. Subsequent analyses were performed for non-smokers and smokers across different OBS levels.

**Results:**

OBS was inversely associated with PRISm in all models (all *p*-values < 0.001). In subsequent analyses, the odds ratio (*OR*) for PRISm increased sequentially from non-smokers with high OBS, to non-smokers with low OBS, to smokers with high OBS, and to smokers with low OBS (dose–response *p*-values in all models ≤ 0.003); smokers with low OBS exhibited the highest PRISm incidence in the fully adjusted model (*OR* = 1.83, 95% *CI* = 1.27–2.64, *p* = 0.001).

**Conclusion:**

A lower OBS was associated with an increased incidence of PRISm, particularly among smokers, suggesting that OS may play a pivotal role in this relationship.

## 1 Introduction

Unlike chronic obstructive pulmonary disease (COPD), preserved ratio impaired spirometry (PRISm) is characterized by the absence of airflow obstruction with reduced lung function (1). PRISm is defined as a normal or preserved ratio of forced expiratory volume in 1 s (FEV_1_) to forced vital capacity (FVC; FEV_1_: FVC ≥ 0.7), coupled with an FEV_1_ below 80% of the predicted value (1). With a global prevalence ranging from 5 to 20%, PRISm is associated with an increase in respiratory symptoms (1, 2) and a higher risk of adverse clinical outcomes (3–5). A prospective cohort study with a 4.5-year follow-up reported that 50% of PRISm patients progressed to COPD, while 15% reverted to normal lung function (6). Given its reversible nature, PRISm represents a promising target for preventive strategies that can avert a poor prognosis. While previous studies have identified several risk factors for PRISm, including smoking status, abnormal body mass index (BMI), and age (1, 2, 7), the pathogenesis remains unclear, and interventions to reverse PRISm and restore normal lung function are limited.

Oxidative stress (OS), a term first coined by Helmut Sies in 1985, refers to an imbalance in which prooxidants outnumber antioxidants, leading to tissue damage and subsequent organ dysfunction (8). Previous research has confirmed that OS induced by smoking contributes to the decline in lung function among smokers (9). Numerous exogenous factors influence OS levels in the body; for instance, physical activity and certain nutrients, such as vitamin C, calcium, and selenium, act as antioxidants, whereas obesity, smoking status, and excessive alcohol consumption are prooxidant factors (10). The oxidation balance score (OBS), which incorporates 20 dietary and lifestyle-related pro-oxidant and antioxidant components, is a validated tool for assessing the overall impact of exogenous factors on OS status, with higher OBS values indicating a greater capacity to resist oxidative damage (10). A recent study found that a higher OBS score was associated with a lower incidence of COPD (11). However, few studies have investigated the association of OBS with PRISm. We therefore aimed to investigate the relationship between OBS and PRISm. Furthermore, considering that smoking is a risk factor for PRISm and that smoking-induced OS impairs lung function, we hypothesize that a healthy diet and lifestyle, by acting as antioxidant strategies, may mitigate the impact of smoking on PRISm risk. Thus, to test this hypothesis, we compared the risk of PRISm in non-smokers and smokers with different OBS levels.

## 2 Methods

This study was exempt from additional ethical review, as it involved secondary analysis of a public database.

### 2.1 Study population

This study utilized data from the National Health and Nutrition Examination Survey (NHANES), conducted by the National Center for Health Statistics (NCHS), to assess the health and nutritional status of the US population. NHANES procedures include interviews, physical examinations conducted in mobile examination centers, and laboratory tests. Further details regarding the research protocol and written informed consent are available on the NHANES website.^1^

**Figure 1:**
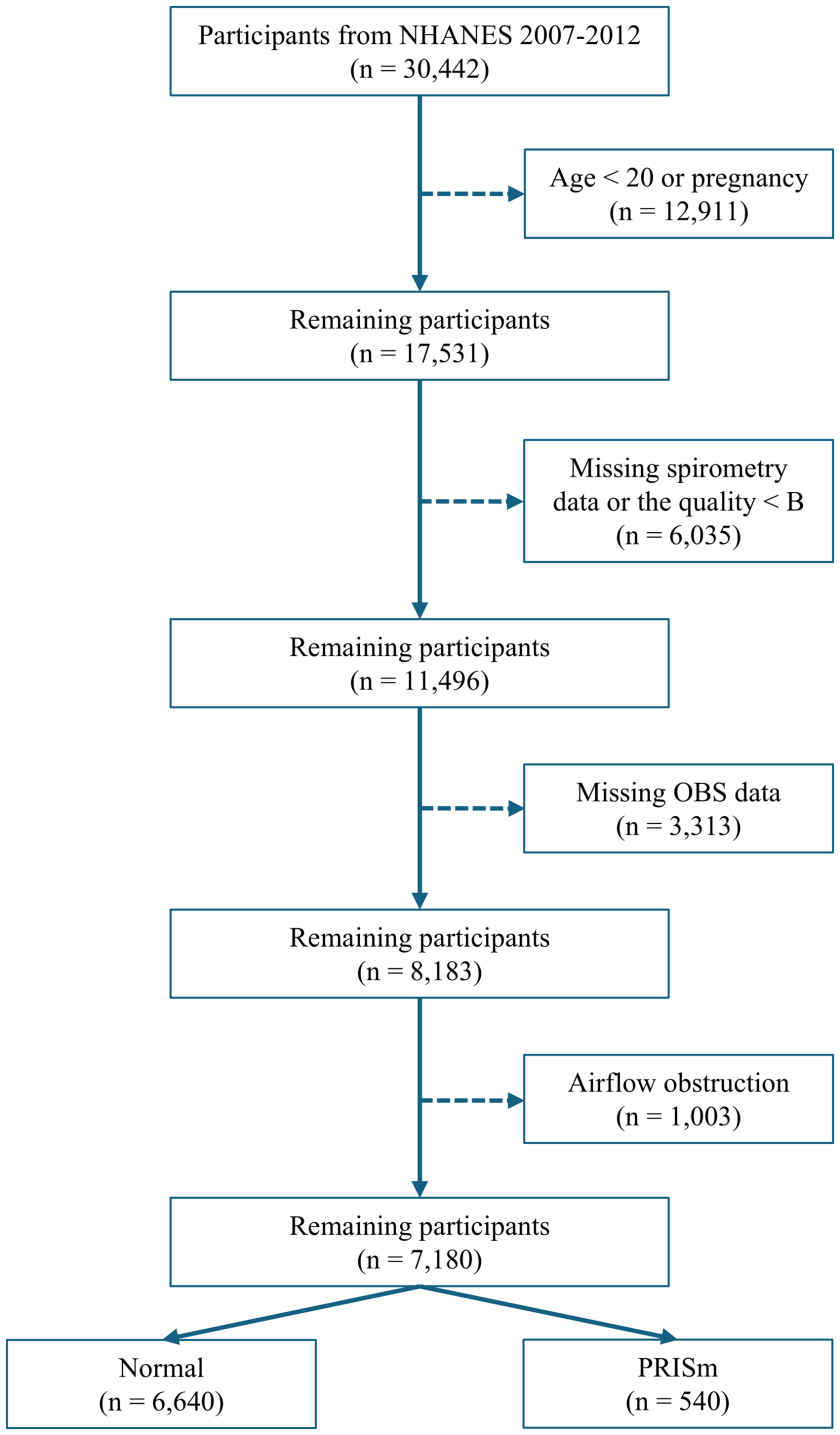
Flowchart for selecting participants.

Data from three consecutive NHANES cycles (2007–2008, 2009–2010, and 2011–2012), encompassing 30,442 participants with available spirometry data, were used. Participants were excluded based on the following criteria: age < 20 years old, pregnancy, lack of spirometry data, or data quality below grade B (B refers to meeting American Thoracic Society data collection standards: three acceptable curves and two reproducible curves, with two observed values within 150 mL), missing data on diet and lifestyle factors necessary for OBS calculation, and evidence of airflow obstruction (FEV_1_: FVC < 0.70; Figure 1).

### 2.2 Definition of PRISm

PRISm was defined as FEV_1_: FVC ≥ 0.7 and FEV_1_ < 80% of the predicted value. FEV_1_ and FVC data were obtained from the SPX dataset. Predicted FEV_1_ values were calculated using the new “race-neutral” Global Lung Function Initiative (GLI) equation (12).

### 2.3 OBS calculation

Based on data from the interview questionnaire—which includes computer-assisted personal interviewing and audio computer-assisted self-interviewing systems, both programmed with built-in consistency checks to reduce data entry errors, and with the computer-assisted personal interviewing system using online help screens to assist interviewers in defining key terms used in the questionnaire—physical examination, and laboratory tests, 20 components—comprising 16 dietary nutrients and 4 lifestyle factors—were categorized into 5 prooxidants and 15 antioxidants for the OBS calculation, following gender stratification (Supplementary Table 1). Alcohol consumption was categorized into non-drinkers, light drinkers (female < 15 g/day versus male < 30 g/day), and heavy drinkers (female ≥ 15 g/day versus male ≥ 30 g/day), with points assigned as 2, 1, and 0, respectively. The remaining 4 prooxidants were assigned 2, 1, and 0 points for the low, medium, and high tertile groups, respectively; conversely, all 15 antioxidants were assigned 0, 1, and 2 points (10).

### 2.4 Covariates

Age (categorized as young adult < 25 years, adult < 45 years, middle-aged < 65 years, and aged ≥ 65 years), gender, ethnicity, poverty–income ratio (categorized as low ≤ 1.3, middle ≤ 3.5, and high > 3.5), education level (classified into less than 9th grade, 9–11th grade, high school grade, some college or AA degree, and college graduate or above), marital status (defined as “not single” including “living with a partner” and “married,” or “single”), BMI (categorized as underweight < 18.5, normal < 25, overweight < 30, and obese ≥ 30), smoking status (defined as non-smoker): “not smoked 100 cigarettes in life,” former smoker (smoked at least 100 cigarettes in life but not smoking now,” and current smoker), and drinking frequency (defined as non-drinker, < 5 days per month, 5–10 days per month, and ≥10 days per month) were analyzed as covariates.

### 2.5 Statistical analysis

#### 2.5.1 Baseline characteristics

The baseline characteristics of PRISm cases and normal controls were presented, with continuous variables presented as medians and categorical variables presented as percentages. Pearson’s chi-squared and Wilcoxon rank-sum tests were used for categorical and continuous variables, respectively.

#### 2.5.2 Relationship between OBS and PRISm

Binary logistic regression analyses were conducted to assess the relationship between OBS or quartile OBS and PRISm in model 1. These results were then adjusted for age, gender, and ethnicity in model 2 and further adjusted for economic conditions, education, marital status, BMI, smoking status, and alcohol consumption in model 3. The results are expressed as odds ratio (*OR*) and 95% confidence interval (*CI*).

#### 2.5.3 Association of smoking in addition to OBS with PRISm

Smoking status was redefined as non-smokers (smoking^−^) and current smokers (smoking^+^)—former smokers were classified as non-smokers. A new OBS was recalculated using 19 components, excluding the cotinine component, and categorized as either higher OBS (OBS^+^) or lower OBS (OBS^−^) based on the 50th percentile. Participants were then reclassified into four mutually exclusive groups based on the combination of OBS and smoking status: smoking^−^/OBS^+^, smoking^−^/OBS^−^, smoking^+^/OBS^+^, and smoking^+^/OBS^−^. Finally, *OR*s for PRISm were calculated for the four groups.

#### 2.5.4 Sensitivity analysis

To address concerns about the instability of the PRISm definition caused by the use of different predicted-FEV_1_ equations, we replaced the GLI equation with the NHANES III equation and reran all analyses (13).

Continuous variables are represented as medians with interquartile ranges, while categorical variables are represented by counts and percentages. Pearson’s chi-squared and Wilcoxon rank-sum tests were used for categorical and continuous variables, respectively. A binary logistic regression model was used for the primary analysis, and various models adjusting for different covariates were used. Trend tests were conducted to evaluate the dose–response effect when the exposure factor was treated as an ordinal variable. All statistical analyses were performed using R version 4.4.1. A two-tailed *p*-value of < 0.05 was considered statistically significant.

## 3 Results

### 3.1 Baseline characteristics by PRISm status

Among the 7,180 participants, 540 (7.5%) were classified as PRISm cases, while 6,640 (92.5%) served as controls. Significant differences were observed between the PRISm and control groups in terms of age, ethnicity, household income, education level, marital status, BMI, smoking status, and alcohol consumption. The median OBS was 18 in the PRISm group versus 21 in the control group (*p*-value of < 0.001; Table 1).

**Table 1:** Participants’ characteristics at baseline.

Characteristic	Normal *N* = 6,640^1^	PRISm *N* = 540^1^	*p*-value^2^
OBS	21 [15, 26]	18 [12, 23]	<0.001
Age			<0.001
Young adult	842 (12.68%)	42 (7.78%)	
Adult	2,947 (44.38%)	186 (34.44%)	
Middle aged	2,183 (32.88%)	241 (44.63%)	
Elderly	668 (10.06%)	71 (13.15%)	
Gender			0.2
Female	3,258 (49.07%)	281 (52.04%)	
Male	3,382 (50.93%)	259 (47.96%)	
Ethnicity			<0.001
Non-Hispanic White	3,123 (47.03%)	111 (20.56%)	
Non-Hispanic Black	1,057 (15.92%)	327 (60.56%)	
Mexican American	1,156 (17.41%)	18 (3.33%)	
Other Hispanic	730 (10.99%)	21 (3.89%)	
Other ethnicities	574 (8.64%)	63 (11.67%)	
Household Income			0.005
Low	1,774 (28.81%)	152 (30.40%)	
Middle	2,140 (34.75%)	201 (40.20%)	
High	2,244 (36.44%)	147 (29.40%)	
Not recorded	482	40	
Education			<0.001
Less than 9th grade	512 (7.72%)	25 (4.63%)	
9–11th grade	875 (13.19%)	78 (14.44%)	
High school grade	1,394 (21.01%)	146 (27.04%)	
Some college or AA degree	2,055 (30.97%)	192 (35.56%)	
College graduate or above	1,800 (27.12%)	99 (18.33%)	
Not recorded	4	0	
Marital Status			<0.001
Not single	4,031 (60.74%)	277 (51.39%)	
Single	2,606 (39.26%)	262 (48.61%)	
Not recorded	3	1	
BMI			<0.001
Normal	1,940 (29.22%)	111 (20.56%)	
Underweight	64 (0.96%)	13 (2.41%)	
Overweight	2,318 (34.91%)	136 (25.19%)	
Obesity	2,318 (34.91%)	280 (51.85%)	
Smoking			0.013
Non-smoker	3,899 (58.76%)	290 (53.70%)	
Former smoker	1,408 (21.22%)	114 (21.11%)	
Current smoker	1,329 (20.03%)	136 (25.19%)	
Not recorded	4	0	
Drinking			<0.001
Non-drinker	683 (12.12%)	89 (20.18%)	
1–5 drinks/month	3,342 (59.29%)	260 (58.96%)	
5–10 drinks/month	616 (10.93%)	37 (8.39%)	
10+ drinks/month	996 (17.67%)	55 (12.47%)	
Not recorded	1,003	99	

1 Data are presented as the median [25th–75th percentile] or n (%).

2 The *p*-value was calculated using the Wilcoxon rank-sum test or Pearson’s chi-squared test.

PRISm, preserved ratio impaired spirometry; OBS, oxidation balance score; BMI, body mass index.

### 3.2 Association between OBS and PRISm

In model 1, OBS was inversely associated with PRISm (*OR* = 0.95, 95% *CI* = 0.93–0.96, *p* < 0.001). This inverse association remained significant after adjusting for age, gender, and ethnicity in model 2 (*OR* = 0.96, 95% *CI* = 0.95–0.98, *p* < 0.001) and remained stable in the fully adjusted model 3 (*OR* = 0.97, 95% *CI* = 0.95–0.98, *p* < 0.001). Compared to the lowest quartile (Q1), the highest quartile (Q4) of OBS was associated with a significantly lower incidence of PRISm across all models (all *p*- and *p*-trend values < 0.001). In the fully adjusted model 3, the *OR* was 0.53 (95% *CI* = 0.37–0.75; Table 2).

**Table 2:** Binary logistic regression analysis of the relationship between OBS and PRISm.

Exposure	Model 1	Model 2	Model 3
	*OR* (95% *CI*) *p-*value	*OR* (95% *CI*) *p-*value	*OR* (95% *CI*) *p-*value
OBS	0.95 (0.93–0.96)	0.96 (0.95–0.98)	0.97 (0.95–0.98)
	< 0.001	< 0.001	< 0.001
OBS (Quartile)			
Q1 (4–15)	Reference	Reference	Reference
Q2 (16–21)	0.73 (0.58–0.90)	0.81 (0.64–1.02)	0.82 (0.62–1.07)
	0.004	0.075	0.15
Q3 (22–26)	0.57 (0.44–0.72)	0.68 (0.52–0.88)	0.76 (0.56–1.02)
	< 0.001	0.003	0.073
Q4 (27–36)	0.37 (0.28–0.48)	0.51 (0.38–0.67)	0.53 (0.37–0.75)
	< 0.001	< 0.001	< 0.001
*P* for Trend	< 0.001	< 0.001	< 0.001

Model 1, unadjusted; Model 2, adjusted for age, gender, and race; Model 3, adjusted for age, gender, ethnicity, economic conditions, education, marital status, BMI, smoking, and drinking.

### 3.3 Relationship between smoking status with OBS and PRISm

The smoking^−^/OBS^+^ group served as the reference and was compared to the other three mutually exclusive groups: the smoking^−^/OBS^−^ group, the smoking^+^/OBS^+^ group, and the smoking^+^/OBS^−^ group. Logistic regression analyses demonstrated that the smoking^−^/OBS^−^ group was a significant risk factor for PRISm in all models (all *p*-values < 0.009); in model 3, the *OR* was 1.43 (95% *CI* = 1.10–1.88). Similarly, the smoking^+^/OBS^−^ group exhibited a positive association with PRISm across the three models (all *p*-values ≤ 0.001); in model 3, the *OR* was 1.83 (95% *CI* = 1.27–2.64). Interestingly, smoking^+^/OBS^+^ was positively correlated with PRISm in models 1 and 2 (both *p*-values < 0.05), but this statistical significance was not observed in the fully adjusted model 3. Most notably, a significant dose–response relationship was observed across the groups, with OR values progressively increasing from the smoking^−^/OBS^+^ group (reference group, *OR* = 1.00), followed by the smoking^−^/OBS^−^ and smoking^+^/OBS^+^ groups, and finally reaching the highest in the smoking^+^/OBS^−^ group (in all three models, for the dose–response trend, all *p*-values ≤ 0.003; Table 3).

**Table 3:** PRISm odds ratio for the four mutually exclusive groups based on OBS and smoking status.

	*OR*	95% *CI*	*p-*value	*p* for trend
Model 1				< 0.001
Smoking^−^ OBS^+^		Reference		
Smoking^−^ OBS^−^	1.94	1.57–2.40	< 0.001	
Smoking^+^ OBS^+^	1.57	1.09–2.22	0.013	
Smoking^+^ OBS^−^	2.26	1.71–2.97	< 0.001	
Model 2				< 0.001
Smoking^−^ OBS^+^		Reference		
Smoking^−^ OBS^−^	1.57	1.26–1.97	< 0.001	
Smoking^+^ OBS^+^	1.51	1.02–2.19	0.034	
Smoking^+^ OBS^−^	1.96	1.46–2.64	< 0.001	
Model 3				0.003
Smoking^−^ OBS^+^		Reference		
Smoking^−^ OBS^−^	1.43	1.10–1.88	0.009	
Smoking^+^ OBS^+^	1.50	0.96–2.31	0.070	
Smoking^+^ OBS^−^	1.83	1.27–2.64	0.001	

Model 1, unadjusted; Model 2, adjusted for age, gender, and ethnicity; Model 3, adjusted for age, gender, ethnicity, economic conditions, education, marital status, BMI, smoking, and drinking.

### 3.4 Sensitivity analysis

The sensitivity analysis aligned with the primary analysis, suggesting that the results are robust to the instability of the PRISm definition when using different equations for predicted FEV_1_ (Supplementary Tables 2, 3).

## 4 Discussion

We used OBS as an index of overall OS status induced by exogenous factors and investigated its relationship with PRISm, along with the consideration of smoking status. Our findings indicate that co-exposure to OS and smoking is associated with a higher risk of PRISm. If these relationships were causal, a healthy diet and lifestyle could potentially aid in the recovery of smokers with PRISm, serving as a form of antioxidant therapy.

### 4.1 Therapy for PRISm

Although PRISm, with its high prevalence, is associated with clinically significant respiratory symptoms and poor prognosis (1–5), therapeutic options for PRISm are limited. Consequently, respiratory medications are often used improperly (14, 15). In the SPIROMICS study, 42% of PRISm patients used bronchodilators, and 23% used inhaled glucocorticoids (15). In contrast, in the COPDGene study, 20% used respiratory medication, and this high rate was related to worse symptoms (14). However, the RETHINC study, a randomized controlled trial (RCT), demonstrated that inhaled dual bronchodilator therapy is ineffective in relieving symptoms (16). Since smoking is the primary cause of PRISm, smoking cessation remains a primary goal; nevertheless, interventions to alleviate symptoms and reverse PRISm to normal lung function remain necessary. The lack of effective treatments for PRISm is partly due to its unclear pathogenesis.

### 4.2 OS in PRISm

OS is implicated in a myriad of diseases, including atherosclerosis (17), Alzheimer’s disease (18), cancer (19), and COPD (20). It can be categorized into two types based on its etiological role: primary versus secondary, or direct versus indirect (8). Smoking-induced OS in COPD is a classic example of a secondary cause contributing to disease progression. Specifically, cigarette smoking, a rich source of oxidants, induces OS, which, in turn, inhibits α1-antitrypsin and neutrophil elastase in the lungs—the pathological hallmark of COPD (21). Furthermore, OS triggers inflammation and cell death-and fibrosis-related pathological cascades, thereby accelerating the pathological progression of COPD (22). Moreover, OS-related gene polymorphisms serve as “susceptibility factors” in the progression to COPD (9). Given its role in COPD, OS is likely also involved in PRISm. Our study found an association between OBS and PRISm, thereby supporting this assumption and illuminating potential mechanisms. Further experimental studies on the pathogenesis are necessary to obtain conclusive evidence.

### 4.3 Antioxidant strategies against OS in PRISm

With COPD, potential antioxidant strategies to reduce OS include thiol-based antioxidants, antioxidant mimetics, peroxidase inhibitors, mitochondria-targeted antioxidants, and dietary antioxidants. However, the majority of therapeutic targets remain either unsatisfactory or have not been sufficiently studied, with the exception of nutritional antioxidants (8, 22). Diets rich in prooxidants are linked to poor lung function and contribute to the development of COPD (23), whereas a Mediterranean diet rich in antioxidants is associated with a lower risk of COPD (24). L-ascorbic acid (vitamin C) and *α*-tocopherol (vitamin E) are well-known dietary antioxidants, and their role in OS has been extensively studied (25–27). Given that a single nutrient cannot represent the combined effect of all OS-related nutrients, the OBS provides a comprehensive assessment of OS-related nutrients and lifestyle, reflecting the levels of OS induced by exogenous factors (10). In other words, the OBS can estimate how well a certain diet and lifestyle can withstand OS. We found that, compared with smoking^−^/OBS^+^, smoking^+^/OBS^+^ had a higher PRISm risk, and the *OR* increased further in smoking^+^/OBS^−^. This trend suggests that a healthy diet and lifestyle may offer antioxidant therapy for PRISm. However, more RCTs focusing on the impact of a healthy diet and lifestyle on PRISm are essential.

### 4.4 Strengths

This study is the first to demonstrate an association between PRISm and OS, offering novel insights into the pathogenesis of PRISm and guiding future research. Moreover, the variation in PRISm incidence across the four mutually exclusive groups combining smoking status and OBS suggests potential intervention strategies, underscoring the role of a healthy diet and lifestyle in the management of PRISm. NHANES data, with their large sample size and rigorous survey design, ensured the reliability of our findings. Sensitivity analysis using different predicted FEV1 equations further corroborated our findings.

### 4.5 Limitations

This study has several limitations. First, the incidence of PRISm may have been overestimated due to the use of prebronchodilator spirometry data, although the impact of bronchodilator administration on PRISm incidence has not been fully established. Second, as an index, the OBS can only indirectly reflect exogenous OS status, while the endogenous part was not evaluated in our study. This may limit our interpretation of OS in PRISm. Third, as this is a cross-sectional study, logistic regression cannot establish causality. Fourth, although multiple models were used to adjust for several common confounding factors, some unmeasurable confounders remain inevitable and may bias our conclusions.

## 5 Conclusion

A higher OBS was associated with a lower incidence of PRISm, suggesting that OS may play a key role in this relationship. A healthy diet and lifestyle may function as an antioxidant therapy for PRISm, particularly among smokers.

## Data Availability

The original contributions presented in the study are included in the article/Supplementary material, further inquiries can be directed to the corresponding author.

## Footnotes

^1^ https://wwwn.cdc.gov/Nchs/Nhanes/
