# Supplementary material for: Association between the oxidative balance score and preserved ratio impaired spirometry in US adults: NHANES 2007–2012
Source: Front Nutr. 2025 Aug 6;12:1551237. doi: 10.3389/fnut.2025.1551237 (PMC12364635; doi:10.3389/fnut.2025.1551237)
Supplement: Supplementary file 1 [file Table_1.docx]

Supplementary Table 1: Oxidative balance score assignment.

|  |  | Male | | | Female | | |
| --- | --- | --- | --- | --- | --- | --- | --- |
| OBS components | Property | 0 | 1 | 2 | 0 | 1 | 2 |
| Dietary OBS components |  |  |  |  |  |  |  |
| Dietary fiber (g/d) | A | <12.9 | 12.9-21.4 | ≥21.5 | <10.8 | 10.8-17.4 | ≥17.5 |
| Carotene (RE/d) | A | <232.0 | 232.0-499.9 | ≥500.0 | <201.0 | 201.0-419.2 | ≥419.3 |
| Riboflavin (mg/d) | A | <1.72 | 1.72-2.67 | ≥2.68 | <1.34 | 1.34-1.9 | ≥2.0 |
| Niacin (mg/d) | A | <22.4 | 22.4-33.6 | ≥33.7 | <15.7 | 15.7-23.5 | ≥23.6 |
| Vitamin B_6_ (mg/d) | A | <1.7 | 1.7-2.6 | ≥2.7 | <1.2 | 1.2-1.8 | ≥1.9 |
| Total folate (mcg/d) | A | <327.0 | 327.0-523.9 | ≥524.0 | <252.0 | 252.0-399.9 | ≥400.0 |
| Vitamin B_12_ (mcg/d) | A | <3.5 | 3.5-7.2 | ≥7.3 | <2.5 | 2.5-5.1 | ≥5.2 |
| Vitamin C (mg/d) | A | <33.4 | 33.4-103.1 | ≥103.2 | <30.6 | 30.6-88.8 | ≥88.9 |
| Vitamin E (ATE) (mg/d) | A | <5.6 | 5.6-9.9 | ≥10.0 | <4.5 | 4.5-7.7 | ≥7.8 |
| Calcium (mg/d) | A | <717.0 | 717.0-1198.6 | ≥1198.7 | <601.0 | 601.0-971.9 | ≥972.0 |
| Magnesium (mg/d) | A | <263.0 | 263.0-381.9 | ≥382.0 | <206.0 | 206.0-301.9 | ≥302.0 |
| Zinc (mg/d) | A | <9.7 | 9.7-15.2 | ≥15.3 | <7.1 | 7.1-10.6 | ≥10.7 |
| Copper (mg/d) | A | <1.1 | 1.1-1.5 | ≥1.6 | <0.9 | 0.9-1.2 | ≥1.3 |
| Selenium (mcg/d) | A | <100.7 | 100.7-151.8 | ≥151.9 | <71.2 | 71.2-107.7 | ≥107.8 |
| Total fat (g/d) | P | ≥107.5 | 67.5-107.4 | <67.5 | ≥78.0 | 49.0-77.9 | <49.0 |
| Iron (mg/d) | P | ≥19.2 | 12.4-19.1 | <12.4 | ≥14.6 | 9.5-14.5 | <9.5 |
| Lifestyle OBS components |  |  |  |  |  |  |  |
| Physical activity (MET-minute/week) | A | <1680.0 | 1680.0-5879.9 | ≥5880.0 | <893.3 | 893.3-2879.9 | ≥2880.0 |
| Alcohol (g/d) | P | ≥30 | 0-29 | None | ≥15 | 0-14 | None |
| Body mass index (kg/m^2^) | P | ≥30.0 | 25.7-29.8 | <25.7 | ≥30.9 | 25.0-30.8 | <25.0 |
| Cotinine (ng/mL) | P | ≥2.65 | 0.03-2.64 | <0.03 | ≥0.12 | 0.02-0.11 | <0.02 |

Abbreviations: OBS, oxidative balance score; A, antioxidant; P, prooxidant; RE, retinol equivalent; ATE, alpha-tocopherol equivalent; MET, metabolic equivalent.
