# Supplementary material for: Association between the oxidative balance score and preserved ratio impaired spirometry in US adults: NHANES 2007–2012
Source: Front Nutr. 2025 Aug 6;12:1551237. doi: 10.3389/fnut.2025.1551237 (PMC12364635; doi:10.3389/fnut.2025.1551237)
Supplement: Supplementary file 2 [file Table_2.docx]

Supplementary Table 2. Sensitive analysis of binary logistic regression for the relationship between OBS and PRISm, using NHANES III equation.

| Exposure | Model 1 | Model 2 | Model 3 |
| --- | --- | --- | --- |
|  | *OR* (95 % *CI*)  *p-*value | *OR* (95 % *CI*)  *p-*value | *OR* (95 % *CI*)  *p-*value |
| OBS | 0.96 (0.95-0.97) | 0.96 (0.95-0.98) | 0.98 (0.96-0.99) |
|  | < 0.001 | < 0.001 | 0.005 |
| OBS (Quartile) |  |  |  |
| Q1 (4-13) | Reference | Reference | Reference |
| Q2 (14-19) | 0.80 (0.62-1.02) | 0.80 (0.62-1.03) | 0.93 (0.70-1.24) |
|  | 0.077 | 0.086 | 0.6 |
| Q3 (20-24) | 0.64 (0.48-0.84) | 0.64 (0.49-0.85) | 0.71 (0.51-0.99) |
|  | 0.001 | 0.002 | 0.043 |
| Q4 (25-35) | 0.49 (0.36-0.65) | 0.53 (0.39-0.71) | 0.67 (0.47-0.94) |
|  | < 0.001 | < 0.001 | 0.022 |
| *P* for Trend | < 0.001 | < 0.001 | 0.008 |

Note: Model 1, unadjusted; Model 2, adjusted for age, gender, and race; Model 3, adjusted for age, gender, race, economic conditions, education, marital status, BMI, smoking, and drinking.
