# Supplementary material for: Association between the oxidative balance score and preserved ratio impaired spirometry in US adults: NHANES 2007–2012
Source: Front Nutr. 2025 Aug 6;12:1551237. doi: 10.3389/fnut.2025.1551237 (PMC12364635; doi:10.3389/fnut.2025.1551237)
Supplement: Supplementary file 3 [file Table_3.docx]

Supplementary Table 3. Sensitive analysis of PRISm odds ratio for the four mutually exclusive groups based on OBS and smoking status, using NHANES III equation.

|  | *OR* | 95 % *CI* | *p-*value | *p* for trend |
| --- | --- | --- | --- | --- |
| Model 1 |  |  |  | < 0.001 |
| Smoking^−^ OBS^+^ |  | Reference |  |  |
| Smoking^−^ OBS^−^ | 1.60 | 1.27-2.03 | < 0.001 |  |
| Smoking^+^ OBS^+^ | 1.16 | 0.75-1.73 | 0.5 |  |
| Smoking^+^ OBS^−^ | 2.22 | 1.65-2.97 | < 0.001 |  |
| Model 2 |  |  |  | < 0.001 |
| Smoking^−^ OBS^+^ |  | Reference |  |  |
| Smoking^−^ OBS^−^ | 1.50 | 1.18-1.91 | < 0.001 |  |
| Smoking^+^ OBS^+^ | 1.24 | 0.80-1.87 | 0.3 |  |
| Smoking^+^ OBS^−^ | 2.33 | 1.72-3.14 | < 0.001 |  |
| Model 3 |  |  |  | < 0.001 |
| Smoking^−^ OBS^+^ |  | Reference |  |  |
| Smoking^−^ OBS^−^ | 1.46 | 1.11-1.93 | 0.007 |  |
| Smoking^+^ OBS^+^ | 1.35 | 0.83-2.15 | 0.2 |  |
| Smoking^+^ OBS^−^ | 2.18 | 1.51-3.14 | < 0.001 |  |

Note: Model 1, unadjusted; Model 2, adjusted for age, gender, and race; Model 3, adjusted for age, gender, race, economic conditions, education, marital status, BMI, smoking, and drinking.
